# Horses (*Equus caballus*) facial micro-expressions: insight into discreet social information

**DOI:** 10.1038/s41598-023-35807-z

**Published:** 2023-05-27

**Authors:** Claude Tomberg, Maxime Petagna, Lucy-Anne de Selliers de Moranville

**Affiliations:** Faculty of Medicine, Université Libre de Bruxelles, 808, Route de Lennik, CP 630, 1070 Brussels, Belgium

**Keywords:** Behavioural ecology, Social evolution, Animal behaviour, Cognitive neuroscience

## Abstract

Facial micro-expressions are facial expressions expressed briefly (less than 500 ms) and involuntarily. Described only in humans, we investigated whether micro-expressions could also be expressed by non-human animal species. Using the Equine Facial action coding system (EquiFACS), an objective tool based on facial muscles actions, we demonstrated that a non-human species, *Equus caballus*, is expressing facial micro-expressions in a social context. The AU17, AD38 and AD1 were selectively modulated as micro-expression—but not as standard facial expression (all durations included)—in presence of a human experimenter. As standard facial expressions, they have been associated with pain or stress but our results didn’t support this association for micro-expressions which may convey other information. Like in humans, neural mechanisms underlying the exhibit of micro-expressions may differ from those of standard facial expressions. We found that some micro-expressions could be related to attention and involved in the multisensory processing of the ‘fixed attention’ observed in horses’ high attentional state. The micro-expressions could be used by horses as social information in an interspecies relationship. We hypothesize that facial micro-expressions could be a window on transient internal states of the animal and may provide subtle and discreet social signals.

## Introduction

Described so far only in humans, micro-expressions are fleeting facial expressions resulting from short-lasting contraction of facial muscles^1^. While some divergences among authors do exist, the maximal duration of said micro-expressions has been generally fixed at less than 500 ms^2^. Contrasting with macro-expressions, facial micro-expressions are not the result of voluntary control^3,4^ and are difficult to be intentionally produced or repressed^5^. They may reveal inner emotional states and authentic feelings^6^. According to Ekman^3^, micro-expressions are believed to reflect a person’s true intent. Initially they were described as a fleeting facial expressions exhibited while trying to conceal a genuine emotion^1^ but the ability to detect a true liar based on their facial micro-expression has been contested^7^ and was not very conclusive^8^. Independently of this ability -or inability- to catch a liar, micro-expressions are an interesting tool to detect an individual’s transient emotional and mental states which are barely perceptible to humans^9^.

The facial expressions result from the contraction of muscles attached to the skin of the face^10^. Existing in all vertebrates^11^, these muscles have evolved in mammals to contribute to the facial display of emotions and communicative signals*,* additionally to their feeding and respiratory functions^12^. For example, different species of mammals have the ability to recognize individuals’ face and extract emotional information from conspecific’s facial display^13–16^. Evidence of the social communication function of the facial expressions in nonhuman animals was e.g. shown by the adaptation of the facial expressions according to their potential audience^17,18^*.*

The functions assigned to the humans’ micro-expressions are currently the same as those of any facial expression as they are identical to the standard ones but their short duration renders them out of voluntary control (too short to allow the feedback loops to control voluntarily the muscles activity) and so provides them their specificity, i.e. to give a window on the true feelings of the sender. Yet, apart from the emotion they carry over, the communication function has hardly been studied so far.

To serve as social signal, micro-expressions must be perceived by the observer. Due to the short exposure of micro-expressions, the neural mechanisms underlying their recognition by a perceiver differ from those at play in the recognition of macro-expressions^19^. Yet the question of their supraliminal or subliminal recognition has not been clearly addressed. To recognize micro-expression is hard for a human observer^1^ and this process is modulated by several factors. A recognition rate of less than 40% have been reported^20^ with higher ratings depending of the duration of the micro-expressions and the emotion expressed with a better rate for happiness^21^. Several other factors influence the accuracy of recognition such as the intensity of expression^22^, the age^23^ and personality^24^ of the perceiver and the emotional context^25,26^. Empathy e.g. enhances recognition of micro-expression of anger^27^. Another mechanism involved in the recognition of micro-expressions is facial feedback^28^. It is produced by the involuntarily contraction of facial muscles during perception of another’s facial expression. When facial feedback is temporarily blocked (by e.g. chewing or intentionally refraining mimicry), the accuracy of recognition is usually decreased^29^. Interestingly, facial feedback occurs also in response to expressions of which the perceiver is unaware^30–32^ reinforcing the findings that unseen facial expressions may be reliably processed^33^. Whether this mechanism is at play in human-animal interactions remains to be elucidated as facial movements appear to have evolved to be species-specific^34^.

In our study we used horses (*Equus caballus*) as biological model. They have the ability to generate a wide array of facial expressions^35^; fewer than those of humans^36^, but more than those of e.g. chimpanzees^37^ or dogs^38^. Facial expressions displayed by horses may convey emotions like pain^39^ or emotions of positive valence^40^. Moreover horses’ facial expressions may convey social information^15^. But as highly gregarious species produce a wide variety of facial movements which may function in group cohesion by enhancing communication during conflict management and bonding^41,42^ it could be expected that horses facial expressions could also serve as a communicative tool for social interactions as they are social animals living in a fission–fusion social system similar to humans^43,44^. The level of complexity of their communicative facial behaviours would provide information about evolutionary development of the horses’ behavioural repertoire relative to their social system and ecological factors. One dimension of this complexity is the range of duration of the facial expressions (others dimensions are e.g. the diversity, the intensity and the laterality of the facial displays). This study investigates if horses display facial micro-expressions and if these could be part of an interspecies social signal system.

Like the facial expressions, human micro-expressions can be objectively described using the Facial action coding system (FACS) which is a rigorous taxonomy of facial movements based on anatomical structure of muscles insertion^45^. They are coded as (1) Action Units (AUs) representing a specific movement produced by the contraction of a facial muscle (or set of muscles) or as (2) Action Descriptors (ADs) for more global facial movements where the muscular basis either cannot be precisely identified or is the result of a different muscle set (e.g. deep muscles)^36^.

Adapted FACSs have been recently developed for facial expressions analysis of several species like chimpanzees^37^, dogs^38^ and horses^35^. They use the same coding index based on comparative anatomy with humans. When the movement is the same as that of humans but performed by different muscles or set of muscles, a “1” is added before the AU code. When the movement is executed by the same muscles but produce quite a different movement a letter representing the species is added before the AU (i.e. “H” for horse).

Our study is the first to investigate if facial micro-expressions are specific to humans or if they are shared with other animals species. Here we use the horse (*Equus caballus*) as model and the Equine Facial Action Coding System (EquiFACS)^35^ adapted from human FACS as tool. Horses are an ideal model to investigate micro-expressions because they exhibit complex facial expressions and have abilities into interspecies communication including being sensitive to attentional states of humans^46,47^ and to their cues^48^. This study uses data from a previous experiment which aimed to investigate the dynamics of horses’ cross-species communication with humans. Here we hypothesize that analyzing separately micro-expressions and facial expressions of longer duration could provide information about the animal’s transient internal states which would otherwise be hidden among all the facial expressions combined. Our prediction is that facial micro-expressions could be used by horses as subtle and discreet communicative signals in a horse-human interaction.

## Results

### Facial micro-expressions

Facial expressions as measured by AUs and ADs with a duration below the threshold of 500 ms were found. 100% of the horses expressed micro-expressions and 66.5% of all facial expressions were micro-expressions. 32.6% of the micro-expressions were related to eye closure and lid lowering.

### Characterization of facial expressions

The mean duration of all facial expressions included, was of 202 ms ± 115 ms. Fourteen Action Units and three Action Descriptors were expressed by more than 25% of the horses. The AU24; AU27; AD160; AU16+17; AU122 were expressed by less than 25% of the horses. A variant of the AU18 was expressed by only one horse: a stretching movement of both lips forwards expressed only in micro-mode (0.14 s ± 0.02). In horses the AU18 is mostly seen in the top lip only^35^; here this movement was observed in both lips as generally seen in humans. We also observed a combination of AU16 and AU17 but only one time by one horse and expressed in micro-mode (0.16 s).

**Figure 1:**
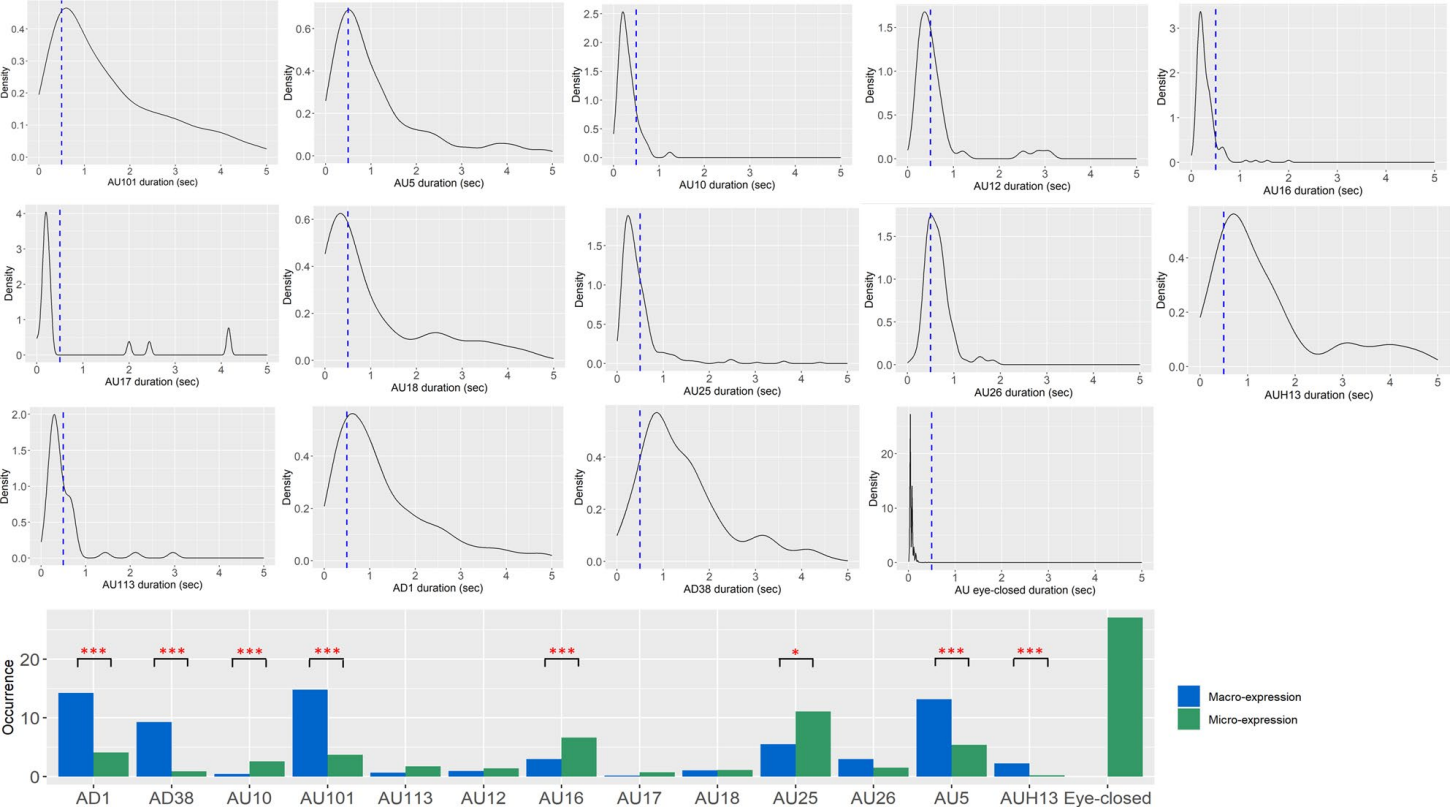
Graphs of Kernel density estimation and bar graph for each Action Unit (AU) and Action Descriptor (AD) expressed by at least 30% of the horses, both conditions included. In the graphs of density estimation, a vertical dotted line was set at 500 ms separating the micro-expression (duration < 500 ms) from the macro-expression (duration > 500 ms). In the bar graph, the respective occurrence of the Action Units and Action Descriptiors as micro- and macro-expression was indicated with statistical significance, p < 0.05 (*), p < 0.01 (**), p < 0.001 (***). Due to the too small number of individuals having expressed the AU17 and the eye-closed in macro-expression, no statistical comparison was calculated for these facial expressions (and no asterisks indicated).

The distribution of the durations of each facial expression exhibit in the two conditions were plotted with Kernel density graphs (Fig. 1). The AU122 and the closed eyes were only expressed in micro-mode (duration shorter than 500 ms). Some facial expressions were significantly more frequently expressed in micro-mode than in macro-mode (duration longer than 500 ms) (Wilcoxon test): AU10, V = 78, p = 0.002, AU16, *V* = 120, p < 0.001, AU 25, *V* = 141, p = 0.02. AU17 is also mainly expressed in micro-mode as only one horse expressed AU17 in macro-mode. Other facial expressions were significantly more frequently expressed in macro-mode than in micro-mode: AU5, *V* = 6, p < 0.001, AU101, *V* = 0, p = 0.005, AUH13, *V* = 1, p = 0.005, AD1, *V* = 5, p < 0.001, AD38, *V* < 0, 0.001.

#### In control condition

Sixty-six percent of all facial expressions were micro-expressions. The AU122 was only expressed in micro-mode. Some facial expressions were significantly more frequently expressed in micro-mode than in macro-mode (Wilcoxon test): AU16, *V* = 55, *p* = 0.006, AU10, *V* = 36, *p* = 0.014. The AU5, *V* = 13, *p* = 0.008, AD1,* V* = 7, *p* = 0.001, AU101, *V* = 0, *p* = 0.001, AD38, *V* = 0, *p* = 0.001, were significantly more frequently expressed in macro-mode and a marginal difference was found for AUH13 with overexpression in macro-mode, *V* = 4, *p* = 0.059. For the AUs 12, 18, 24, 25, 26 and 113, there was no significative differences between micro- and macro-expressions. The AU17 and AU24 couldn’t be properly characterized in control condition as too few were expressed in this condition.

#### In experimental condition

Sixty-seven percent of all facial expressions were micro-expressions. Their mean duration was not significantly different than in control condition except for AU26 as standard facial expression, *V* = 56, *p* = 0.045 (Wilcoxon test) (Table 1). Some facial expressions were expressed by more individuals in experimental condition than in control condition so they were better characterized in experimental condition. This was the case for UA 17 with 3.3 more individuals expressing it in experimental condition with a mean duration of 0.61 s ± 1.25.

**Table 1:** Mean duration of facial expressions in the control and experimental condition.

Micro + Marco						
Action unit	Control condition		Test condition		Comparison	
	Mean duration (s)	Stand dev	Mean duration (s)	Stand dev	Statistic	p
AU101 inner brow raiser	1.66	1.33	1.67	2.01	V = 176	ns
AU5 upper lid raiser	1.2	1.25	1.04	0.9	V = 367.5	ns
AU10 upper lip raiser	0.34	0.22	0.53	0.81	V = 46	ns
AU12 lip corner puller	0.74	0.7	0.57	0.5	V = 16	ns
AU113 sharp lip puller	0.45	0.21	0.57	0.47	V = 30	ns
AUH13 nostril lift	6.5	9.18	4.77	5.33	V = 21	ns
AU16 lower lip depressor	0.43	0.32	0.63	1.46	V = 94	ns
AU17 chin raiser	0.84	1.19	0.62	1.25	V = 6	ns
AU18 lip pucker	0.97	1	1.47	1.66	V = 4	ns
AU122^I^ upper lip curl	0.16	_	0.19	0.023	_	_
AU24^I^ lip presser	0.35	0.24	0.71	1.07	_	_
AU25 lips part	0,62	0.57	0.67	0.81	V = 129.5	ns
AU26 jaw drop	0.58	0.19	0.7	0.78	V = 56	0.045
AU27^I^ mouth stretch	0.42	0.5	1.5	1.61	_	_
AD1 eye white increase	1.31	0.9	1.35	1.45	V = 280.5	ns
AD38 nostril dilatator	2.09	2.05	1.5	1.36	V = 113	ns
AD160^I^ lower lip relax	1.31	1.41	1.08	0.96	_	_
Eye closed	0.06	0.02	0.06	0.026	V = 120	ns

Micro						
Action unit	Control condition		Test condition		Comparison	
	Mean duration (s)	Stand dev	Mean duration (s)	Stand dev	Statistic	p
AU101 inner brow raiser	0.33	0.083	0.38	0.064	t = − 1.65	ns
AU5 upper lid raiser	0.3	0.1	0.29	0.12	t = 0.195	ns
AU10 upper lip raiser	0.22	0.067	0.24	0.12	t = − 0.4995	ns
AU12 lip corner puller	0.34	0.1	0.32	0.14	t = 0.2867	ns
AU113 sharp lip puller	0.31	0.12	0.29	0.1	W = 21.5	ns
AUH13 nostril lift	0.16	_	0.36	0.11	_	_
AU16 lower lip depressor	0.24	0.074	0.24	0.064	t = − 0.305	ns
AU17 chin raiser	0.16	0.08	0.22	0.036	_	_
AU18 lip pucker	0.26	0.078	2.61	1.72	t = − 1.208	ns
AU122^I^ upper lip curl	0.16	_	0.56	_	_	_
AU24^I^ lip presser	0.18	_	0.53	0.18	_	_
AU25 lips part	0.29	0.072	0.27	0.076	t = -0.616	ns
AU26 jaw drop	0.39	0.054	0.38	0.11	W = 18	ns
AU27^I^ mouth stretch	0.135	0.035	0.36	_	_	_
AD1 eye white increase	0.29	0.09	0.32	0.13	t = -0.64	ns
AD38 nostril dilatator	0.31	0.15	0.31	0.134	t = 0.0148	ns
AD160^I^ lower lip relax	0.25	0.15	0.4	_	_	_
Eye closed	0.064	0.017	0.064	0.026	W = 271.5	ns

Macro						
Action unit	Control condition		Test condition		Comparison	
	Mean duration (s)	Stand dev	Mean duration (s)	Stand dev	Statistic	p
AU101 inner brow raiser	2.56	0.95	2.78	2.22	V = 111	ns
AU5 upper lid raiser	2.01	1.26	1.63	0.81	V = 229	ns
AU10 upper lip raiser	0.63	0.2	1.49	1.43	V = 2	ns
AU12 lip corner puller	0.96	0.8	1	0.63	V = 9	ns
AU113 sharp lip puller	0.68	0.035	0.18	0.49	V = 7.5	ns
AUH13 nostril lift	7.41	9.52	5.75	0.16	V = 28	ns
AU16 lower lip depressor	0.79	0.28	2.56	3.39	V = 11.5	ns
AU17 chin raiser	2.22	_	4.16	_	_	_
AU18 lip pucker	2.04	0.45	2.61	1.72	_	_
AU122^I^ upper lip curl	_	_	_	_	_	_
AU24^I^ lip presser	0.52	_	2.2	_	_	_
AU25 lips part	1.18	0.62	1.15	1.03	V = 79	ns
AU26 jaw drop	0.73	0.08	0.98	1.01	V = 37	ns
AU27^I^ mouth stretch	1	_	2.64	_	_	_
AD1 eye white increase	2.21	1.21	2.19	1.5	V = 185	ns
AD38 nostril dilatator	2.45	2.065	1.92	1.35	V = 148	ns
AD160^I^ lower lip relax	2.37	1.21	1.76	_	_	_
Eye closed	_	_	_	_	_	_

Standard facial expression includes micro- and macro-expressions. ^I^Action Units and Action Descriptors expressed by less than 25% of the horses; ^#^Action Units and Action Descriptors expressed by less than 3 horses in one of the two conditions.

The AU122 was only expressed in micro mode. The AU16 (Wilcoxon test), *V* = 105, *p* = 0.001, UA10, *V* = 10, *p* = 0.009, AU25, *V* = 148, *p* = 0.007 and AU17, *V* = 48, *p* = 0.041 were significantly more frequently expressed in micro-mode while the AU5, *V* = 0, *p* < 0.001, AD1, *V* = 6, *p* < 0.001, AU101, *V* = 0, *p* = 0.003, AD38, *V* = 2, *p* < 0.001 and AUH13, *V* = 3, *p* = 0.

### Control versus experimental conditions comparison of facial expressions

**Figure 2:**
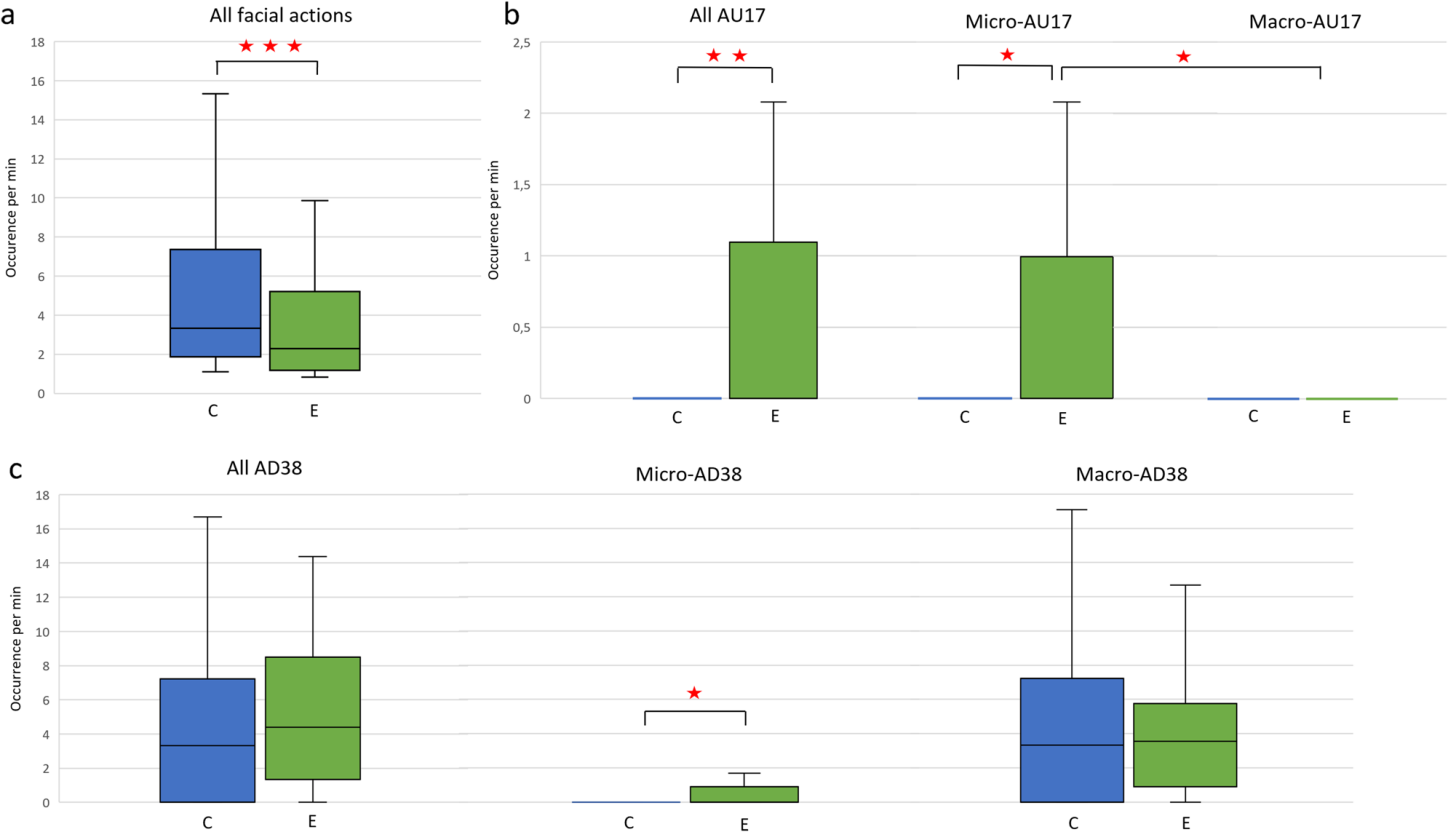
Comparisons of facial expressions occurrences in control (C) and experimental condition (E). This graph illustrates the added value of analyzing the micro-expression separately from the facial expressions all duration combined. (**a**) All facial expressions included (micro- and macro-expressions collected). (**b**) All AU17 -chin raiser- (micro- and macro-expressions included) (left boxplots); comparison of micro-AU17 and macro-AU17 in control and experimental conditions (right boxplots). (**c**) All AD38 -nostril dilatator- (micro-and macro-expressions included), micro-AD38 and macro-AD38 compared in control and experimental conditions. P < 0.05(*), p < 0.01(**), p < 0.001(***).

The horses expressed nine percent less facial expressions (Wilcoxon test), *V* = 8324.5, *p* < 0.001 (Fig. 2a) and four percent less micro-expressions, *V* = 18,910, *p* = 0.001, in experimental than in control condition.

The AU17 was significantly more frequently expressed in experimental than in control condition in both micro- and macro-mode combined, *V* = 3, *p* = 0.024 as well as in micro-mode, *V* = 1,* p* = 0.021 (Fig. 2b). AD38 was not differentially expressed in micro- and macro-mode combined *V* = 96, *p* = 0.6632 but was marginally more frequently expressed in experimental condition, *V* = 1, *p* = 0.059 in micro-mode (Fig. 2c) with an effect size of (Cohen’s d) 0.42. AU12 was marginally more frequently expressed in control condition, *V* = 32, *p* = 0.059 in macro-mode but not in micro- and macro-mode combined, *V* = 47, *p* = 0.23. The AD1 was not significantly more expressed in micro mode in experimental condition than in control condition, *V* = 123.5, *p* = 0.260.

### Control versus experimental conditions comparison of ears positions

**Figure 3:**
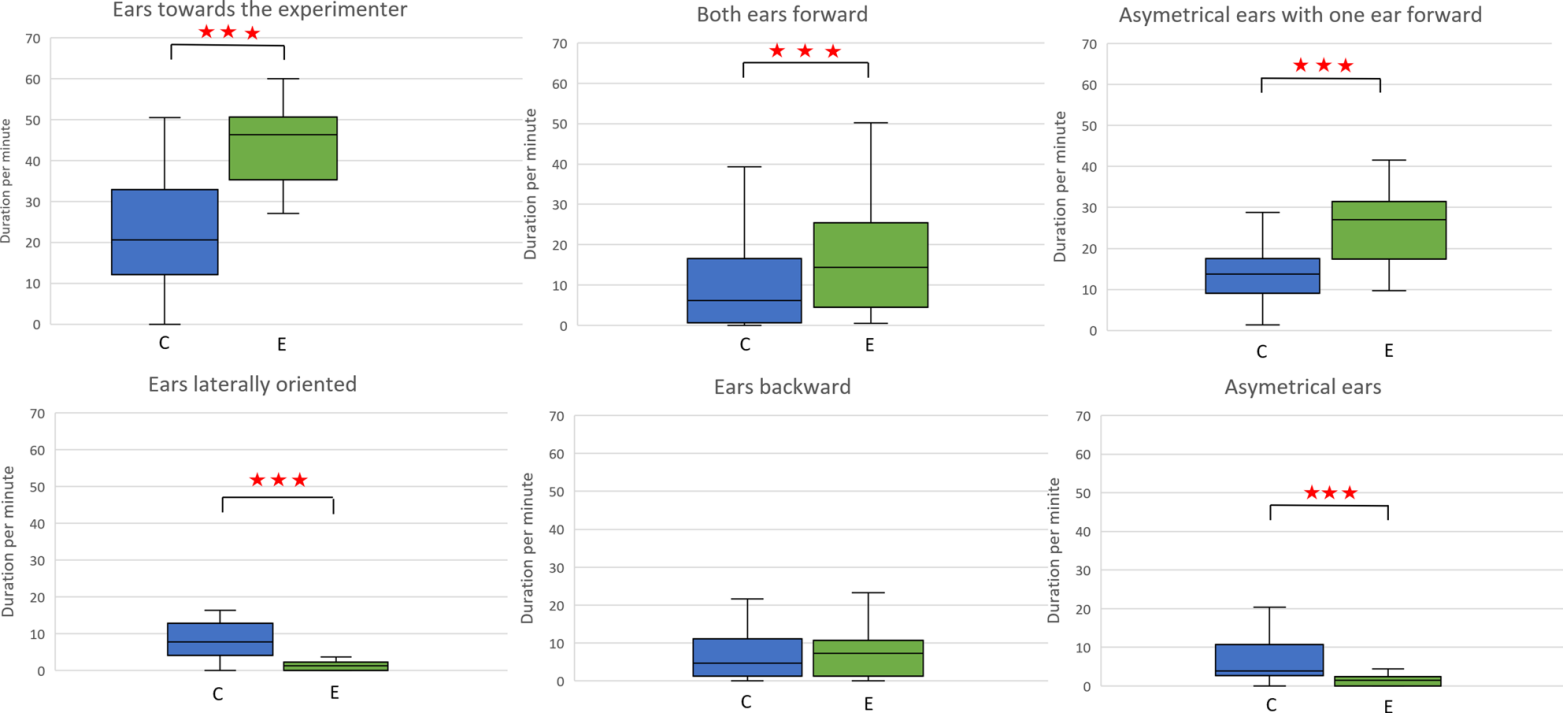
Comparisons of ears orientation in the control (C) and experimental condition (E). p < 0.05(*), p < 0.01(**), p < 0.001(***).

The ears (Fig. 3) were significantly more oriented toward the experimenter’s position in the experimental condition than in the control (Wilcoxon test), *V* = 5, *p* < 0.001. Both ears were significantly more oriented forward (Student test), *t*(21) = 3.916, *p* < 0.001 as well as asymmetrically (with only one ear forward), *V* = 11, *p* < 0.001) in the experimental condition than in the control. The ears were significantly more laterally oriented, *t*(21) = 5.089, *p* < 0.001, in asymmetrical position (one ear forward excluded), *t*(21) = 3.812, *p* < 0.001 and in forward position (excluded toward the experimenter’s position), *t*(21) = 3.006, *p* = 0.007, in the control than in the experimental condition. No significative difference was found for the backward position, *t*(21) = − 0.022, *p* = 0.983.

### Correlation of facial micro-expressions with behavioural index of attention orientation

**Figure 4:**
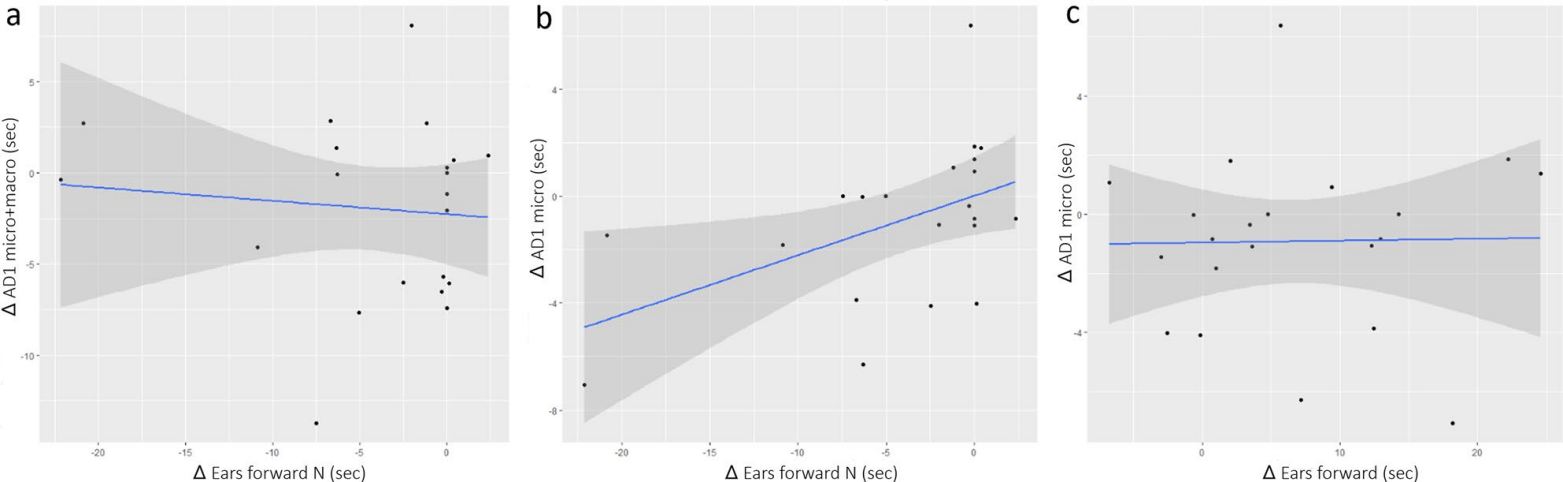
Correlations of AD1 (eye white increase) with ears position. (**a**) Standard AD1 with ears oriented forward in directions other than toward the experimenter (ears forward N). (**b**) Micro-AD1 with ears oriented in directions other than toward the experimenter, (**c**) micro-AD1 with ears oriented forward toward the experimenter.

A significant positive moderate correlation was found between the change in the two ears forward (not oriented toward the experimenter’s position, labeled as “N”) between the experimental (T) and control (C) conditions (∆ ears forwards N) and change in AD1 occurrence in micro-mode (∆ AD1 micro) (Pearson correlation test), *r* = 0.45, *p* = 0.043, but not toward the experimenter’s position (∆ ears forwards) (Fig. 4a, b). This correlation was not found for AD1 both modes combined, *r* = − 0.17, *p* = 0.453 (Fig. 4c).

**Figure 5:**
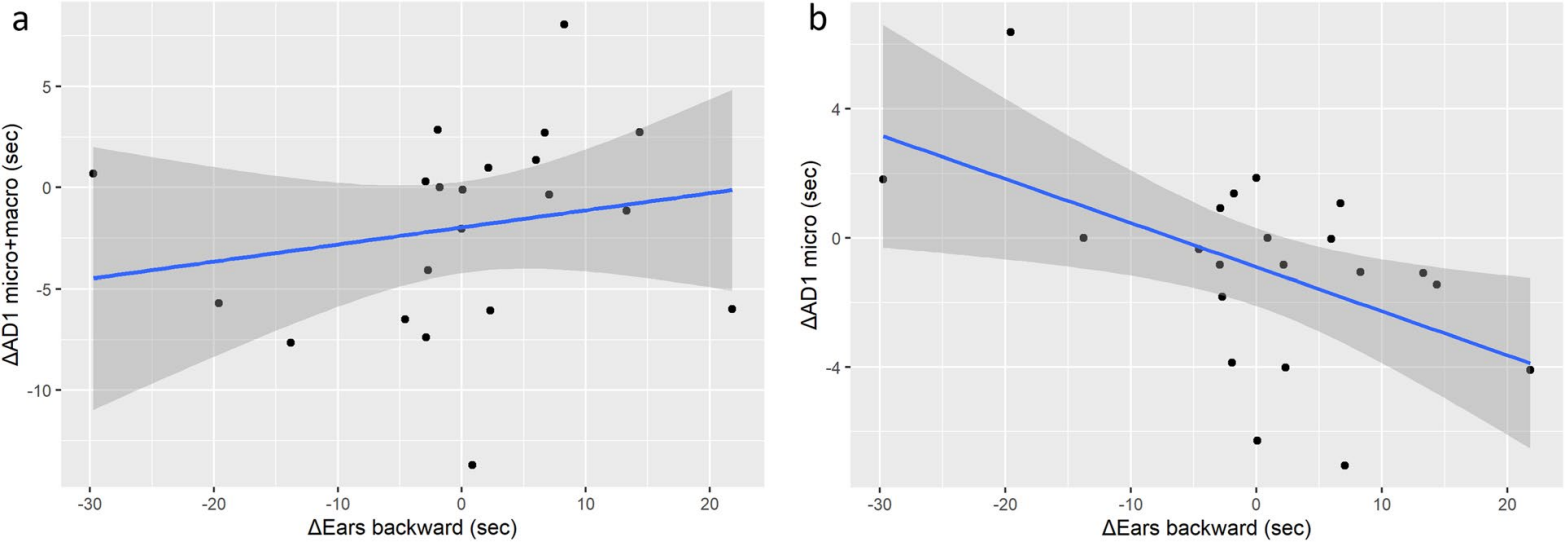
Correlations of AD1 with ears position. (**a**) Standard AD1 with ears oriented backward. (**b**) Micro-AD1 with ears oriented backward.

A significant moderate negative correlation was found between the change in ears oriented backwards (∆ ears backwards) and the change in AD1 occurrence in micro-mode (∆ AD1 micro), *r* = − 0.52, *p* = 0.016 (Fig. 5a). This was not found for all AD1 mode included, *r* = 0.22, *p* = 0.340 (Fig. 5b).

### Association between AU17, AD 38 and AD1 facial expressions

The AU17, AD 38 and AD1 have been associated as facials indicators of pain^39,49^ or stress^50^. We tested the association between these facial expressions. In micro and macro mode combined, they were significantly different (Friedman test), *F*(2,21) = 24.2, *p* < 0.001 and were not rank correlated (Kendall test), *W* = 0.374, *p* = 0.316. In micro-mode, theses facial expressions were differentially expressed, *F*(2,21) = 7.97, *p* = 0.019 and were independent, *W* = 0.374, *p* = 0.316.

### Behavioural measures

Behavioural indicators have been measured in order to evaluate potential stress related differences between the conditions. No significant differences were found between conditions for the body displacements occurrences (Wilcoxon test), *V* = 103.5, *p* = 0.747, the ears movements occurrences (Student test), *t*(21) = 0.725, *p* = 0.476 and the chewing duration, *V* = 103.5, *p* = 0.747. The chewing took part for 20.9% of all behaviours expressed.

Behavioural indicators have also been measured in order to evaluate potential frustration. No significant differences were found between conditions for the forward body displacements occurrences (Friedman test), *F*(2,21) = 0.189, *p* = 0.911 nor for the trampling on, *F*(2,21) = 1.279, *p* = 0.528. No significant correlations were found between the forward body movements and the micro-AU17 (Spearman correlation test), *ρ*  = − 0.037, *p* = 0.87, the micro-AD38, *ρ* = 0.140, *p* = 0.55, or the micro-AD1, *ρ*  = − 0.305, *p* = 0.179 in the experimental condition. No significant correlations were found between the trampling on movements and the micro-AU17, *ρ* = − 0.041, *p* = 0.861, the micro-AD38, *ρ* = 0.181, *p* = 0.432 or the micro-AD1, *ρ* = 0.323,* p* = 0.153 in the experimental condition.

The ears position were coded in order to evaluate the attention orientation of the horses. The orientation of the ears differed significantly from one condition to the other (Friedman test), *F*(2,42) = 33.1, *p* < 0.001 with more forward pinnae orientation (toward the experimenter position) in experimental condition compared to control (Wilcoxon test) *V* = 5; *p* < 0.001 and carrot control condition *V* = 253; *p* < 0.001 and less forward orientation in carrot control condition compared to control *V* = 229; *p* = 0.001.

## Discussion

This study shows that horses are expressing facial micro-expressions and suggests that some of those could be related to attentional state or could be used by horse as social information in an interspecies relationship.

All facial micro-expressions observed were facial expressions described in the EquiFACS so that we didn’t notice any new unknown facial expression. The duration of some facial expressions were displayed in both micro- and macro- ranges while others displayed durations limited to one range. For the facial expressions of expanding duration on both ranges, some of them were significantly more expressed in one of the two ranges. Interestingly, no facial micro-expressions changed from micro to macro ranges or reverse through the conditions: the AU10, AU16, and the AU122 were significantly more expressed as micro-expressions in both conditions and the AU5, AUH13, AU101, AD1 and AD38 were significantly more expressed as macro-expressions in both conditions. The mean durations didn’t change significantly suggesting that it would be an intrinsic feature of each facial expression.

### Effect of the experimenter on attention orientation

Horse’s attention can be monitored by ear position^51–54^. As each horse’s ear pinna can be mobilized by 18 extrinsic muscles^55^, the ears’ position can be fine-tuned to an attentional target and indicate attention orientation^51,54,56^. In our study the horses spend more time with their ears oriented forward toward the position taken by the experimenter when this latter is present compared to when the horse is alone suggesting enhanced attention toward the experimenter and/or the carrot. Yet it was unclear if this increase could be related to the experimenter themselves, the carrot or both. When only the carrot was in front of the horse, the ears were a significantly 64% less forward oriented than in presence of the experimenter. Horses have been shown to increase their attention towards the human experimenter with mere gazes and monitoring in case of a food reward^47^ or when having difficulty reaching food^57^. They relied on humans to solve their problem. In our study the carrot couldn’t be grabbed by the horse. For this purpose the help of the experimenter is required. The higher attention observed towards the experimenter in our study is consistent with previous studies showing that horses may have expectations from humans’ behavior^57,58^. This suggests that the ears rotated forward during test was a reliable indicator of attention towards the experimenter.

### Effect of attention on facial expressions

In our study, the horses expressed significantly less facial expressions and less micro-expressions in presence of the experimenter than when the horse was alone. Horses’ visual attention can include ‘fixed attention’ patterns involving body immobility with orientation of the ears and the eyes towards the stimulus^53^. In humans, neutral face has been correlated with high concentration^59^. Neutral faces are characterized by a neutral positioning of the facial features with no emotion expressed^60^. Yet analysing facial display through automatic recognition of the basic emotions, even if based on combined AUs/ADs, may lead to missing some others facial features as concentration have been also associated in humans with eye narrowing and bringing together the eyebrows^61^. These last were not observed in our data. Maybe eye narrowing during high attention is not an optimal survival strategy for a prey animal as it would decrease the visual field and predator detection. In our study as the horses highly increased their attention toward the experimenter with 75% of the total duration oriented toward the experimenter, the decrease of facial expressions might be related to a high attentional level toward the experimenter. Our results suggest that additionally to the mobilization of the auditory and visual systems^52,56^, fixed attention also includes a modulation of the facial expressions which is coherent with the multisensory processing involved in enhanced attentional processes^62^. Attention modulates multisensory integration processes by organizing the sensory inputs and selecting the allocation cognitive resources in order to help effective perception and cognitive functioning. Our hypothesis is that decreasing facial expressions during high attention allows to free up cognitive resources (which are limited by the brain capacities) and reallocate them to the selection and processing of sensory inputs. Selection of pertinent information among the flow of concurrent sensory inputs which continuously inundate the brain could require the involvement of a high amount of cognitive resources^63^, especially in a survival context where misdetection could have dramatic consequences. This mechanism of selective cognitive resources allocation may be even more prominent in a prey species like horse.

### The micro-AU 17

The AU17 is a facial movement underpinned by contraction of the mentalis muscle which raises the chin^35^ (see in Supplementary files). We found that the AU17 was mainly expressed as a fleeting micro-expression. In humans the AU17 is a part of the expression of sadness^64^. In horses, standard AU17 have been observed in pain^39,49^ but not in stress^65^. Our results showed that the micro-AU17 was significantly more expressed in presence of the experimenter than when the horse was alone. If the micro-AU17 would have expressed pain, it would have been equally expressed in both conditions as our protocol doesn’t inflict pain on the horses. Moreover forward oriented ears have been reportedly displayed by horses when pain-free^66^. In our study the horses spent respectively 53% of the time with their ears forward in control condition and 76% in experimental condition, strengthening the hypothesis that micro-AU17 was not pain-related in our study. Facial expressions could be used flexibly, which means that the same AU or AD can be used in more than one context. In humans, smiling is often expressed in positive environments but also in aversive ones like in covering negative emotions^67^. Our results suggest that in horses, the micro-AU17 may express a different feature than the AU 17 observed in pain.

Due to the experimental conditions of our study where a carrot was placed out of reach but in sight of the horses, we couldn’t exclude some frustration. The literature on equine frustration is limited so far^68^. Body displacements have been identified as behavioural cues of frustration in calves^69^ and dogs^70^. In a Delphi consultation investigating the opinion of equine behavioural experts, consensus agreement suggests among others, increased displacements behaviours, and muscle tension. In our study the horses didn’t increased their body displacements in presence of the carrot nor any trampling on movement. We also didn’t observe significantly more forward movements in the direction of the carrot in a potential attempt to reach it. No correlation was observed between the occurrence of the micro-AU17 and forward displacements or the trampling on movement. These data didn’t support evidence of a relationship between frustration and the micro-AU17. However, behavioural signs of frustration might be variable and not always be obvious to interpret^71^. So that the lack of behavioural indices of frustration in our study doesn’t exclude that the micro-AU 17 would express subtle signs of frustration. Some facial expressions have been associated with frustration in dogs^71^ and cats^72^ but not the AU17. Interestingly, the AU17 was correlated with effort and determination in a frustrating task (a task impossible to solve) in children, but not chimpanzees^73^. If horses but not closer relatives to humans are expressing a facial display involving the same facial muscles as humans in a similar context (a toy in a transparent locked box^73^ versus visible food out of reach in our study) reinforce previous findings that facial movements, including micro-expressions, appear to have evolved to be species-specific^34^.

Social information is information acquired by monitoring others ‘interactions with the environment^74^. Social information could be unintentionally produced like public information^75^ or intentionally produced as communicative signals to inform others^73^. Horses’ facial expressions are visual cues providing a lot of social information^15,76^ and horses primarily communicate visually both with conspecifics^56^ and humans^46^. In our study, as horses both highly increased their attention toward the experimenter and expressed the micro-AU17 only in presence of the experimenter (at the exception of one individual), one can hypothesize that the micro-AU17 may convey a social information. But for a micro-expression to serve as social information, it should be detected by the observer. Horses discriminate global facial expressions of their conspecifics^15^ but whether they detect micro-expressions is still unknown. For naïve humans, it is hard to recognize micro-expressions^6^ but though it is considered almost imperceptible for untrained human observers, micro-expressions have a significant emotional effect on the human perceivers^1^. Whether horses are capable of detecting conspecific’s micro-expressions and how they might be consciously or unconsciously influenced by those should be further investigated. This would confirm if horse’s micro-AU17 is the expression of an emotional state or could also serve as social information and, if so, whether it serves as public information^74^ or is an intentionally communicated signal that changes the receiver’s potential behaviour^77^. Future studies should address the question whether the AU 17 can be interpreted as a kind of unconscious “pointing” with chin towards the carrot or the experimenter with a communicative value, or as a consequence of an experimenter induced change of emotional state.

### The micro-AD 38

The AD 38 is a facial movement underpinned by contraction of a set of muscles which produces the dilatation of the nostril^35^(see in Supplementary files). We found that the global AD 38 (micro- and macro- ranges combined) was not differentially expressed in the control and the experimental condition but the micro-AD38 was selectively marginally more expressed in experimental condition suggesting a modulation in micro range in the presence of the experimenter. Physiologically, nostril dilation is associated with deep breathing and sniffing and the nostril’s opening can change diameter depending on the physiological and psychological state of the animal^56^. For example, nostril dilatation was observed in alert posture^56^ where the cardio-respiratory system is activated to prepare a potential flight^78^. As a facial expression, the AD 38 has been observed in stressful situation^50^. Yet the micro-AD 38 doesn’t seem to be related to the expression of stress. Indeed, indicators of stress like high frequency of body displacements^79,80^ and ears movements^81,82^ were unchanged from one condition to another. The horses showed relaxed tails and no fear reaction suggesting quiet attention^53^. Moreover, the horses performed chewing in both conditions. Chewing has been associated with relaxation^79,83^ and the chewing durations were not significantly different between our experimental conditions suggesting an equal level of comfort in both conditions. These behavioural cues suggest that the increase of micro-AD38 in presence of the experimenter should not be related to stress. Higher levels of attention are not necessarily associated with fear related stress^53^. The AD 38 may also express an affective component of pain^39,49,66^. Pain may increase breathing and induce nasal dilatation^65^. But if micro-AD38 was an expression of pain, a high micro-AD38 should have been expressed in all conditions which was not the case; additionally our conditions didn’t induce pain. It should be noted that up until now most studies addressing the facial expressions of horses have been related to negative emotional valence like pain and stress but little is known about facial expressions of more neutral or positive valence. A recent study suggests that AD38 may be related to positive emotional valence in resting conditions of carriage horses^84^. Nostril dilatation is part of ritualized behaviours expressed in stallions’ interactions and it conveys communicative signals^56^. Nostril dilatation is also involved in auditive^85,86^ and olfactive communication^87,88^. Facial expressions have been suggested to have evolved from ancestral autonomic and protective actions into communicative signals^89,90^. Moreover, the highest social animals have the most facial displays^91^ and horses are highly social animals living in scission-fusion systems^43,44^. In our study, the horses highly increasing their attention toward the experimenter suggests that the micro-AD38 could be a communicative signal which has evolved from a physiological need for increased air intake in case of flight and stress-related situations to social information in high physical activity such as in stallion interactions, becoming, without any more additional physiological need for oxygen, a more subtle signal conveying social information.

### The micro-AD1

The AD1 is a facial movement underpinned by contraction of a set of muscles producing a change in the eye opening allowing more white sclera to be visible^35^ (see in Supplementary files). We found that micro-AD1 (but not global AD1) was correlated with attention orientation. The variation in micro-AD1 expression in presence of the experimenter compared to when the horse is alone, was positively correlated with the change of both ears forward in another direction than the experimenter. The position of both ears forward is indicative of a selective attention process^54^. This suggest an association of the micro-AD1 with selective attention to the horse’s lateral surroundings.

In horses, the AD1 has been associated with stress during transportation or when in experimental social isolation^50^. In sheep, eye aperture was higher during separation from group members^92^. In cows, no increase in AD1 was observed during claw trimming which yet triggered physiological stress responses^93^. An eye white increase has been observed when cows are kept from accessing visible food^94^ but also when exposed to positive expectation^95^ or highly desired food^96^. It has been suggested that the AD1 increase can indicate strong arousal as sympathetic axons innervate the muscle involved in lifting the upper eyelid^97^ and their activation increases the visual field. In our study, as discussed previously, no behavioural indices of stress were noticed in any of the conditions. Due to our experimental condition we couldn’t exclude that frustration could be felt by the horses but as the micro-AD1 was not significantly more expressed in experimental than in control conditions, it suggests that the micro-AD1 should not be related to frustration at least in our experiment. Moreover, if the micro-AD1 had been a facial indicator of stress in our study, the experimenter wouldn’t have induced any stress as the micro-AD1was not correlated with the ears oriented toward the experimenter. The AD1 has also been associated to pain^49^. Again, as previously discussed, no indices of pain were detected in our study. Indeed, the micro-AD1 increase was only observed when the horses oriented their attention on their side. If an increase in micro-AD1 had indicated stress due to pain, this should have been observed for all ear positions which was not the case. We hypothesize that a brief increase in eye white is mechanically related to lateral head turning and could mostly reflect the horses ‘ attempt to improve their visual field in selective attention. In ridden horse, it has been suggested that the presence of eye-white could be a mechanism to gain better forward vision in a strongly flexed head and neck posture^98,99^. This hypothesis is supported by another correlation observed in our study. The change of micro-AD1 expression in presence of the experimenter compared to when the horse is alone, was also negatively correlated with the change of ears oriented backward. The more the horses had their ears oriented backward, the less they disclosed micro-AD1. As the horses have a blind spot right behind them^100^, an improvement of the visual field as manifested by eye-white increase, would not help to detect signals originated from behind the horse. This suggests that when the ears are oriented backward, there is no cooperation between visual and auditory systems. Additionally, if this biomechanically induced micro-AD1 is detected by a perceiver than it could potentially convey public information about the attentional state of the horse. This hypothesis remains to be further investigated.

### General conclusion

In conclusion we show that horses are expressing facial micro-expressions. The mental features that drive them are still unknown. We observed that the AU17, AD38 and AD1 were selectively modulated as micro-expressions but not as standard expressions. Studies that investigated pain- and stress-related global facial expressions have observed significative association between theses 3 facial expressions: AU17, AD 38 and AD1 with some others^49,50^. Yet, we didn’t find any statistical consistency between the expression of theses facial movements nor any behavioural indicators supporting an association of these micro-expressions with stress or pain. There was also no behavioural signs of frustration in our study but since our protocol includes a presumably frustrating situation, we couldn’t exclude that some of the facial displays like the AU17 would express subtle signs of frustration especially as micro-expressions could be fleeting unvoluntary expressions of emotion.

Some authors have suggested that, in humans, neural mechanisms underlying the recognition of micro-expressions differ from those of macro-expressions^6,101^. On the other hand, the neural mechanisms involved in the display of micro-expressions have not yet been specifically addressed. What is known of global facial expressions is that distinct neural structures are involved in voluntary and emotional facial movements and that these two systems are wholly independent from the cortical structures generating them up to the facial nucleus. This organization is probably the reason why genuine emotional facial expression cannot be voluntarily produced^102^. These neural differences and probably some others differences in the intentional motor control that remains to be discovered would support that the micro- and macro-expression of a given AU or AD could differ in the emotional cue or the communicative signal they convey. From the behavioral analysis of our study, the selective modulation of micro-expression within different social contexts suggests that horses’ micro-expressions may also convey other information than the standard facial expressions. We should be aware that the attribution of a single emotion (like a mere stress- or pain-related signal) to a given facial expression could be misleading regarding horses’ displays.

The modulation of these micro-expressions in presence of a human experimenter supports previous data that the horses are sensitive to social context^15,76^. We hypothesize that some of the micro-expressions could be related to attentional state or could be used by horses as social signal in an horse-human relationship. Nevertheless, their classification as communication signals is premature as this study is the first to address the mental features of facial micro-expressions in a non-human animal and their occurrence in different social contexts should be tested. The aim of future research should be to clarify the function of these fleeting micro-expressions, to confirm whether they could serve as social information and, if so, what type of social signal they may convey. It would also be interesting to investigate whether the horses’ ability to perform micro-expressions could be an evolutionary specialization trait shared by other highly social animals.

As limitation of this study, only the right side of face was analyzed. Analyzing both sides raises the complex question of laterality and would require a whole other study focused on the matter. There is not yet a lot of information about asymmetry in animal facial expressions. In humans, the left hemiface is more expressive than the right. The innervation of the muscles of the lower face is contralateral (and bilateral for the muscles of the upper face) so that the muscles of the left hemi-face are receiving motors commands from the right cerebral hemisphere which is more involved in emotional processing than the left one. But in communicative interaction, when looking at the face of another person, and due to the same bias, the visual attention is more oriented toward the right hemi-face of the interlocutor. If applying this to the HAI, the human should be more sensitive to the emotions expressed on the right hemi-face of the horse and the horses should express more on its left hemi-face (of course if the specializations of the horse’s cerebral hemispheres are the same as for humans, which seems (at least for the emotions) phylogenetically the case). Moreover asymmetry could also merge from differences in intensity of the facial expression. This will be even more challenging to analyze and this is another potential bias. Additionally, maybe that for non-emotional signals the bias would not be the same. It has also been suggested that the production of facial displays for “social” emotions would be lateralized to the left hemisphere^102^. All this should be further addressed in forthcoming studies. So, in summary, we hypothesized that analyzing the left side provides a better information on emotional feelings of the sender and analyzing the right side is better to investigate accurately the communicative signals which can provide information on the role of facial micro-expressions in cross-species interaction. We chose to analyze the right side. As second limitation of this study, the coder was trained to use the EquiFacs but was not certified in this skill.

In a more applied perspective, if their function as social signal is confirmed, micro-expressions could potentially provide information on the evolution of subtle features of human-animal communication as some facial expressions have evolved during domestication specifically for communication with humans^103^ or appear to be more expressed in a human than conspecific^104^ context. As micro-expressions are transient fleeting unvoluntary facial displays, they could provide information on the true internal state of the horses. Accurately detecting micro-expressions would also have valuable application potential for horses’ welfare since e.g. as a prey species, they have evolved to behaviourally hide or inhibit the expression of pain as survival strategy^105^.

## Methods

### Participants

Twenty-two horses (*Equus caballus)* (11 mares, 9 geldings and 2 stallions) aged 4–26 years old (mean 14.8 ± 5.6 years) of various breeds participated in the study. Complementary information on each individual can be found as Supplementary table S1 online. The horses were housed in three different stables. No differences in facial expression occurrence were observed between stables, (Rank correlation Kruskall-Wallis test) *H(2)* = 3262, p = 0.1958 . They were led to the fields daily and kept in the stables at night. They had daily contact with their respective human caretakers/riders/owners (being fed, groomed, led between their stall and the fields and for most of them being ridden on a regular basis). They were not food deprived.

### Experimental design

**Figure 6:**
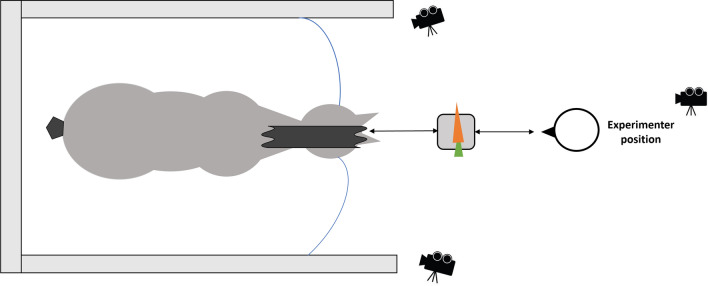
Experimental area.

The experiment took place in the grooming place, an isolated and quiet space in the stables which the horses are familiar with. They were loosely attached on both sides of their halters safely allowing some freedom of movement (Fig. 6).

The same two experimenters, who were unfamiliar with the participating horses, were involved in all recording sessions: one was the timekeeper and recording devices manager while the second engaged in the experimental interactions with the horses.

### Preliminary phase

In a familiarization phase (0–5 min), the horses were handled to the grooming place individually and were accustomed to the recording material. They were introduced first without the equipment which was then introduced progressively with desensitization actions when necessary (waiting with the device at some distance until the horse become familiar with it before placing it at its final experimental position, while one of the experimenter was gently stroking the horse). Once the horse stayed quiet and calm with no more specific interest toward the new device, he was attached and left alone. This was evaluated in consultation by all the experimenters (all familiar with horses) whom checked the following behaviours: no frightened immobility, no tension in the facial muscles nor in the body muscles, quite horizontal position of the neckline or slightly elevated (but no high position like in high attention fixation), no sign of attention specifically oriented toward the new device including head, eyes and ears pointed toward it. Then the experiment would start.

### Experimental series

This study is a part of a more global experiment which aimed to investigate (1) the dynamics of horses’ social cross-species communications with humans and (2) if some behaviours can be classified as intentional relying on criteria established by Townsend et al. 2017^106^. This experiment included two control conditions and seven experimental conditions in which we investigated if horses adapt their behaviours according to the attentional state of the human experimenter. One of these control conditions (named “control condition” in this manuscript) and one of the conditions (named “experimental condition” in this manuscript) were used for micro-expression analysis. For an improved data discussion, the second control condition (“carrot control” condition) was additionally coded but only for attentional behaviours. We selected as behavioural index of attention the ears positions as they are a good indicator of attention orientation^51–54^ and this provided interesting features to discuss the respective attentional investment of the horses relative to the carrot and/or the experimenter. In the control condition, the horse would be left alone for one minute. Then, in the “carrot control” condition, the experimenter came to the subject with a carrot in hand, let the horse smell it then put it on a high stool placed in front of the horse, clearly visible but out of reach for him. Then the experimenter went out. In the experimental conditions, the experimenter entered the experimental space and stood in front of the horse at 0.25 m behind the stool (Fig. 1). The experimenter took a different position at each experimental condition. He stands in front of the horse either (1) attentively, (2) inattentively, (3) with the eyes closed or (4) he turns his back to the horse with his head straight or (5) 1/4 turned or (6) he stands with his body ¾ turned of the horse with his head straight or (7) 1/4 turned. The experimental condition where the experimenter looked attentively at the horse was selected for micro-expression analysis as it was the best and simplest condition to observe a potential horse/human communication without additional variables. The experimenter maintained the position without moving for 1 min and then went out.

A brief vocal signal (“GO”) given by the timekeeper marks the recordings and indicates to the experimenter the onset/offset of test condition.

### Data collection and processing

All tests were recorded by three cameras: a computer camera recording the full stage and two cameras (JVC GZ-V500BE and JVC GZ-EX515BE) focused respectively on the left and right sides of the horses’ head with an angle of 45° to the horse’s body and at a distance of 2 m of the head (Fig. 1). The recordings of the right lateral camera were used to analyze facial expressions.

The videos were analyzed with BORIS software (for Windows Portable v.7.9.8)^70^ on a frame by frame mode at a rate of 25 images/sec. If necessary, a preliminary visual inspection of the videos at a slow rate was performed to ensure the correct identification of facial expression. The data was analyzed by a naïve coder to whom the different conditions were not explained.

The videos were coded in accordance with the Equine Facial Action Coding System (EquiFACS)^35^. MP was the coder, she wasn’t certified in EquiFacs but was trained before coding the data and has a lifetime experience with horses. The onset and offset of the observed Action Units (AU) and Action descriptors (AD) were recorded allowing calculation of duration and frequency. Additionally, two eyelid movements were coded as follows: lowering of the upper lid and eyes closed. The ear positions coding was adapted from Reefman et al.^71^ as lateral (pinna opening oriented perpendicular to the body axis), forward (pinna opening oriented towards the front at an angle of more than 60° from the perpendicular) or backward (pinna opening towards the back at more than 60°from the perpendicular) and asymmetric position (when the two ears were differentially oriented). The asymmetric positions with one ear forward oriented according to the previous criterion were separately coded from all other asymmetric positions. In the data analysis the toward experimenter ear position includes both the two ears forward and at least one ear forward oriented (including asymmetric position with one ear forward and the situations when only one ear forward was visible), the two positions being mutually exclusive. The global duration of only one ear visible was of 7.4% in control condition and 7.5% in test condition. The global duration of both ears invisibility was 2.9% in control condition and 0.7% in test condition. Additionally, body displacement occurrences^79^ (coded as any forward, backward or lateral displacement of at least one step), ear movement occurrences and chewing durations, were measured as potential behavioural indicators of stress.

### Statistical analysis

First, we kept only the image sequences where each AU, AD and ear movement was identifiable over its complete duration. Then we made sure enough duration of visible data was available for each subject. We kept footage in which the horses were visible for minimum 35% of the total recording. Only 5 out of 88 videos had less than 50% analysable recording time. For the control/test comparison, one individual was removed as outlier values were observed. The outlier detection was based on the median plus or minus 2.5 times the Median Absolute Deviation (MAD)^72^. As it is not clear whether this individual truly expressed a lot more than the others or whether this was caused by a bias resulting from normalization of low visibility data (36,7%), we chose to conservatively remove this individual form data analysis.

Normality tests of Schapiro-Wilk were performed to test the normality of all data sets. Then pairwise comparisons between the control and test positions were made using either the parametric Student -test or the non-parametric Wilcoxon signed-rank test with an error risk of first type of 5%. The same tests were performed to compare the occurrences in micro- and macro-mode. For the AU or AD expressed by less than 3 individuals in a given condition, no statistical comparison was calculated as a test of normality cannot be performed with less than 3 values. Correlations were calculated with the parametric Pearson or non-parametric Spearman correlation tests. The Rank correlation Kruskall-Wallis test was used to verify that there were no differences in data between the stables. Statistical computing was performed using RStudio version 4.0.3 (2020-10-10).

Univariate kernel density estimations with gaussian Kernel function were calculated using Stata/SE 16.0 software in order to provide a visual distribution of the durations of each AU and AD.

### Ethical considerations

This study was approved by the Animal Ethic Committee from the University of Liège and was not considered as an experiment in legal terms. The authors complied with the ARRIVE guidelines and regulations. Owners of the horses gave consent prior to participation.

## Supplementary Information

Supplementary Video 1.

Supplementary Video 2.

Supplementary Video 3.

Supplementary Video 4.

Supplementary Video 5.

Supplementary Table S1.

## Data Availability

The raw data and analysis of this study are available from the corresponding author on request.
