# Supplementary material for: Horses (Equus caballus) facial micro-expressions: insight into discreet social information
Source: Sci Rep. 2023 May 27;13:8625. doi: 10.1038/s41598-023-35807-z (PMC10224940; doi:10.1038/s41598-023-35807-z)
Supplement: Supplementary file 6 — Supplementary Table S1. [file 41598_2023_35807_MOESM6_ESM.docx]

**Horses (*Equus caballus*) facial micro-expressions: insight into discreet social information**

**Extended data**

**Supplementary table S1 :** List of the subjects and their complementary information

| **Name** | **Gender*** | **Age (years)** | **Breed**** | **Stables***** | **Discipline** | **Diet** |
| --- | --- | --- | --- | --- | --- | --- |
| Rhapsodie | F | 24 | Han | C | Dressage | Fields & box, retired |
| Dauerfroh | F | 15 | Han | C | Dressage | Fields & box, retired |
| Scarlett | F | 19 | Han | C | Dressage | Fields & box, retired |
| Safana | F | 18 | Ar | C | Dressage | Fields & box, retired |
| Mahony | F | 24 | Ar | C | Dressage | Fields & box, retired |
| Spirit | S | 26 | Ar | C | Dressage | Fields & box, retired |
| Flink | S | 5 | Han | C | Dressage | Fields & box, working |
| Beauty | F | 13 | Ar/Spa | M | Dressage | Fields & box, working |
| Pistache | G | 17 | Hal | M | Leisure | Fields & box, working |
| Lili | F | 4 | We | M | Leisure | Fields & box, working |
| Cascada | F | 10 | SBS | M | Jumping | Box, working |
| Audhela | G | 13 | SBS | M | Jumping | Box, working |
| Altaïr | F | 8 | PaH | M | Leisure | Fields & box, working |
| Ulysse | G | 12 | SBS | M | Leisure | Fields & box, working |
| Velours | G | 13 | SBS | M | Leisure | Fields & box, retired |
| Fench | G | 16 | ¼ Co+¼ Spa+½ Lu | E | Leisure, dressage | Fields & box, retired |
| Delicado | G | 12 | Lu | E | Leisure | Fields & box, working |
| Ouma | G | 14 | Han | E | Dressage | Fields & box, working |
| Alegre | G | 20 | Spa | E | Dressage | Fields & box, working |
| Orée | F | 13 | Han | E | Leisure | Fields & box, working |
| Allstar | G | 15 | Han | E | Dressage | Fields & box, working |
| Karma | F | 15 | Han | E | Dressage | Fields & box, working |

*G: Gelded males, S: Stallions, F: Females

** Ar: Arabian; Spa: Spanish; Han: Hanoverian; Hal: Haflinger; We: Welsch; SBS: Belgian Warmbood ; Co: Connemara; Lu: Lusitano; PaH: Paint Horse

***C: Chaimont stables, M: Mirland stables and E: Eole stables
